# Enhanced Broadband Acoustic Absorption in Commercial Foam via Multiwall Carbon Nanotube‐Induced Pore Reconstruction

**DOI:** 10.1002/advs.202501898

**Published:** 2025-03-24

**Authors:** Jinkui Xiong, Jinlong Liu, Wengui Lin, Yifei Li, Longchao Liao, Mingfu Wen, Guisheng Zhong, Xiaodong Niu, Longshi Rao, Quan Wang, Bin Bao, Qingxian Liu

**Affiliations:** ^1^ Department of Mechanical Engineering Shantou University Shantou Guangdong 515063 China; ^2^ Department of Mechanics and Aerospace Engineering Southern University of Science and Technology Shenzhen Guangdong 518055 China; ^3^ Department of Civil and Environmental Engineering Shantou University Shantou Guangdong 515063 China; ^4^ College of Engineering Eastern Institute of Technology Ningbo Zhejiang 315000 China; ^5^ School of Mechanical Engineering and Automation Harbin Institute of Technology Shenzhen Guangdong 518055 China; ^6^ Intelligent Manufacturing Key Laboratory of Ministry of Education Shantou University Shantou Guangdong 515063 China; ^7^ Shantou Key Laboratory for Intelligent Equipment and Technology Shantou University Shantou Guangdong 515063 China

**Keywords:** acoustic absorption, carbon nanotubes, composite pores, energy dissipation, resonant effect

## Abstract

Noise pollution is an urgent environmental issue that leads to a series of adverse effects on human physical and mental health. Porous materials with rationally designed micropores or channels can effectively absorb noise across wide frequency ranges, making them a well‐established candidate for mitigating acoustic propagation. However, common porous materials with a singular pore structure face a trade‐off between acoustic absorption efficiency and thickness. Herein, this challenge is significantly mitigated by reconstructing the pore structure of commercial melamine foam using multiwall carbon nanotubes (MWCNTs). The melamine/MWCNTs foam exhibits multiscale composite pores, high porosity, and increased specific surface area while preserving the shape and thickness of the initial melamine foam. Due to increased energy dissipation from the porous structure and the resonance effect of MWCNTs, the 10 mm thick composite porous absorber exhibits an average absorption coefficient of ≈70% from 1300 to 6000 Hz, representing a 196.5% increase compared with that of initial melamine foam. The reconstructing pore structure by loading MWCNTs is a simple and general method for improving the acoustic absorption coefficient. It can be extended to other complex morphologies or material systems, offering significant application potential in noise control, acoustic instruments, and architectural design.

## Graphical abstract

A simple to multiscale structure transition happens when a commercial porous foam is filled with multiwall carbon nanotubes with high aspect ratio. For an acoustic absorber with a porous material, the loading of multiwall carbon nanotubes can significantly improve its specific surface area while preserving its thickness and high porosity, thus enables high acoustic absorption and wide applications.

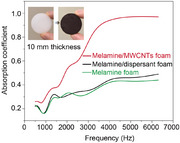

## 1 Introduction

Modern industry has significantly improved living standards but also led to substantial noise pollution. Industrial machinery, high‐speed trains, aircraft, and even household appliances such as air conditioners and washing machines are potential sources of noise that can harm human physical and mental health.^[^ ^1^, ^2^ ^]^ As a result, effective noise control is crucial for protecting human hearing and improving quality of life. Current advancements in noise control technology primarily focus on optimizing the structure of sound sources and suppressing sound waves propagation. Among these, it is widely recognized that vibrations from the sound source are the primary cause of noise. Once the sound source ceases to vibrate, the noise will disappear simultaneously. Optimizing mechanical structures or incorporating damping materials are direct and effective strategies for reducing vibration and noise.^[^ ^3^, ^4^ ^]^ However, once the mechanical structure is finalized, noise reduction at the sound source becomes difficult. Consequently, it becomes necessary to design and manufacture materials with superior noise suppression properties to reduce noise propagation. This remains an important and pressing research topic.^[^ ^5^, ^6^ ^]^

Acoustic absorption materials are mainly categorized into two types: resonant absorbers and porous absorbers. The resonant absorber is equivalent to the parallel connection of multiple Helmholtz resonators, where acoustic energy is dissipated through internal resonance effects.^[^ ^7^, ^8^, ^9^, ^10^ ^]^ The absorbers can achieve high absorption performance for low‐frequency sound by constructing a matched resonance structure. However, resonant absorbers typically exhibit a narrow absorption bandwidth and limited machinability, making it challenging to meet the demands of applications in large venues such as theaters, concert halls, stations, airports, and similar settings.^[^ ^11^ ^]^ In terms of porous absorbers, the principle of absorption is well understood: sound energy is converted into heat energy and dissipated through air‐pore wall friction, viscous resistance, and thermal conduction. This mechanism has been proven to be effective for noise absorption and reduction.^[^ ^12^, ^13^, ^14^ ^]^ Meanwhile, common porous materials can be produced on a large scale and at low cost, making them an economically viable option for commercial acoustic absorbers. However, according to the dissipation theory, the dissipation force is a linear function of the flow rate, and the dissipative power is the product of this force and the flow rate. As a result, the dissipation power exhibits a quadratic relationship with frequency.^[^ ^15^ ^]^ Therefore, common porous materials do not effectively absorb noise, especially in the <2 kHz range (the primary band of urban noise).^[^ ^16^, ^17^ ^]^ Increasing the thickness of the porous materials can enhance noise dissipation capability by extending the propagation path to increase the interaction between sound waves and the material.^[^ ^18^ ^]^ However, this approach significantly increases the material's volume and mass, severely limiting application flexibility. To address this issue and further enhance the absorption efficiency of porous absorbers, various approaches have been explored, including modifying pore distribution, incorporating piezoelectric materials, and integrating vibrational sheets. For example, J. Y. Tang et al. developed a porous fibrous sponge with a gradient structure by using multi‐step multijet blend electrospinning and double cross‐linking. This gradient porous structure presented an increased acoustic absorption under a wide‐band frequency;^[^ ^19^ ^]^ Yao et al. utilized piezoelectric PVDF and single‐walled CNTs to construct a composite foam as a sound absorber, achieving a high absorption coefficient of 50% at a low frequency;^[^ ^20^ ^]^ Gao et al. employed ultra‐thin graphene to construct macro‐acoustic foam, achieving facile and scalable production with significant practical implications for noise control.^[^ ^21^ ^]^ Chen et al. reported a semi‐open cellular structure by incorporating graphene oxide (GO) and functionalized carbon nanotubes, which exhibit ≈100% enhancement over a band gap compared to the pure foam.^[^ ^22^ ^]^ However, these strategies often involve complex preparation steps, toxic reagents, and exhibit limited scalability or mechanical stability. Therefore, developing efficient porous absorbers that possess broadband acoustic absorption, high mechanical properties, and high universality remains a significant challenge.

Herein, we propose a composite porous absorber by reconstructing the pore structure of commercial melamine foam with high aspect ratio MWCNTs. This method not only transforms the simplex large pores of the initial foam into multiscale composite pores while preserving the material's high porosity and thickness, but also significantly increases its specific surface area. These structural features ensure that the sound waves penetrate deeply into the material rather than mere surface reflection, significantly enhancing their propagation path and increasing the probability of sound energy dissipation. Additionally, ultrafine MWCNTs with a high aspect ratio may experience strong vibrations when their natural frequencies match those of the incident sound waves. Both factors contribute to the effective dissipation of sound energy, thereby enhancing the absorption efficiency of the composite foam. The composite foam achieves an average absorption coefficient of 70% across the frequency range of 1300–6300 Hz at 10 mm thickness, representing a 196.5% increase compared to the initial melamine foam. It should be noted that the method exclusively utilizes water as the solvent to obviate the requirement for toxic and harmful reagents, ensuring a safe and environmentally friendly manufacturing process. Furthermore, the direct reconstitution of existing porous materials exhibits high adaptability to substrate shape or raw material, making it highly suitable for industrial production and noise control applications.

## 2 Result and Discussion

**Figure 1:**
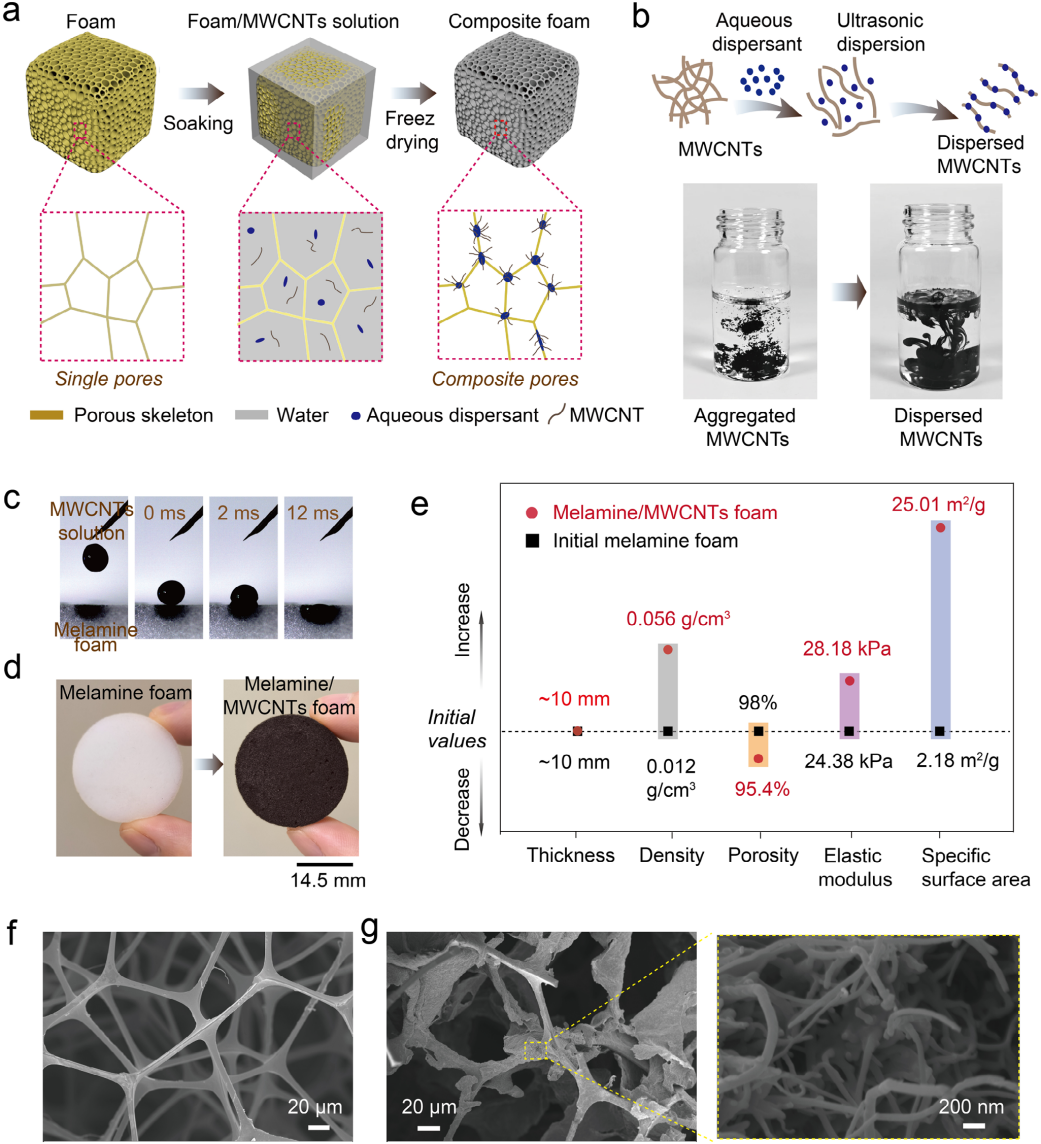
Preparation and structures of the melamine/MWCNTs foam. a) Schematic of the fabrication process of the composite foam, which involves soaking the MWCNTs dispersion in a melamine foam followed by freeze‐drying. The resulting structure diagram is directly demonstrated below each step. b) Schematic of the MWCNTs dispersion by adding dispersant and ultrasonic treatment. c) A spontaneous infiltration process of the MWCNTs solution into a melamine foam. d) Photographs of the initial melamine foam and the melamine/MWCNTs composite foam. e) Structural comparison between the melamine foam before and after loading MWCNTs, focusing on the thickness, density, porosity, elastic modulus, and specific surface area. f) SEM image of the initial melamine foam. g) SEM images of the melamine/MWCNTs composite foam, along with a local magnification that highlights MWCNTs accumulation.

The composite foam was fabricated through immersing a commercial melamine foam in the MWCNTs dispersion and subsequent freeze drying. After removing the solvent (water), the melamine foam's initial simple pore structures transformed into multiscale composite structures due to MWCNT incorporation. This fabrication process and structural change are shown in **Figure** **1**a. During this process, the distribution of MWCNTs within the foam plays a critical role in determining the final structure of the melamine/MWCNTs foam. However, MWCNTs tend to agglomerate due to their high specific surface area, extremely high aspect ratio, and strong van der Waals forces (Figure S1, Supporting Information). To address this challenge, the primary objective is to achieve a stable and homogeneous dispersion of MWCNTs in the solvent. Herein, we employed an aqueous dispersant and ultrasonic assistant to disperse MWCNTs in water. The dispersant coats the surface of the MWCNTs, offering remarkable surfactant properties and a barrier effect to prevent the agglomeration and sedimentation of the MWCNTs, as illustrated in Figure 1b. Experimental results show that the MWCNTs solution with dispersant remains ink‐like over the long term, whereas the solution without dispersant exhibits visible granules due to MWCNT agglomeration (Figure 1b). This strongly evidences the effective dispersion of MWCNTs in water. Moreover, due to the strong hydrophilic nature of the melamine and significant capillary force in microscale pores,^[^ ^23^ ^]^ MWCNTs solution can spontaneously infiltrate into the melamine foam's pores upon contact. This penetration process is completed within 12 ms, as illustrated in Figure 1c. Such a highly efficient process ensures the saturation loading of the MWCNTs solution within the melamine foam, eliminating treatment blind spots. Finally, the water in the MWCNTs‐infused foam is removed by sublimation during freeze‐drying, yielding a multiscale composite porous framework.

This method of reconstructing pores of the commercial melamine foam exhibits several significant characteristics. Externally, the geometry of the porous foam remains largely unchanged after loading MWCNTs, only the color changes to black (see Figure 1d). The thickness (initial ≈10 mm) exhibits a slight increase of 0.5%, which is negligible considering the measurement errors. In terms of porosity, the melamine/MWCNTs composite foam retains a high value of 95.4%, indicating only a 2.25% reduction compared with that of the initial foam of 97.8%. High porosity is crucial for the penetration of acoustic waves into the material, enhancing the opportunity for acoustical absorption. Additionally, high porosity could result in a lower elastic modulus according to the Gibson and Ashbury theory.^[^ ^24^ ^]^ As a result, the elastic modulus of the melamine/MWCNTs foam still presents a low value of 28.18 kPa due to its high porosity. Although it shows a 13.48% increase compared to the initial melamine foam's modulus of 24.38 kPa, the composite foam still maintains high compressible deformability to accommodate various application scenarios. However, the specific surface area of the foam significantly increases from 2.18 to 25.01 m^2^ g^−1^ after loading MWCNTs, which is essential for enhancing the interaction between acoustic waves and the pore walls. Although the density of the composite foam significantly improves due to the increased mass from loading MWCNTs and dispersant, it is only 0.056 g cm^−3^ attributed to its high porosity, which is an order of magnitude lower than that of common woods. These experimental results are visually presented in Figure 1e, which suggests this pore reconstruction method offers a valuable strategy for optimizing the internal structure of porous materials while preserving their shape, thickness, and porosity. Moreover, such a simple process can be scaled up by using an improved freeze‐drying process, as exemplified by a 10 × 25 cm melamine/MWCNTs composite membrane (Figure S2, Supporting Information), exhibiting highly suitable for industrial production.

The microtopography of the melamine foam before and after loading MWCNTs are observed by scanning electron microscope (SEM), and the related images as shown in Figure 1f. Initial melamine foam features a large number of open pores characterized by large size and simple geometries, along with a smooth skeletal surface. These open pores form interconnected channels that provide transport pathways and loading space for the MWCNTs solution, preventing localized accumulation and ensuring homogeneous distribution of MWCNTs within the material. Given the presence of dispersant in the MWCNTs solution, it is essential to investigate its impact on the structure of the composite foam. SEM image reveals that the dispersant mainly coats the skeletal surface without significantly altering pore morphology (Figure S3, Supporting Information). After loading the MWCNTs, a complex composite pore structure emerges, with pore size ranging from ≈135 um to nanoscale due to the accumulation of high aspect ratio MWCNTs, as shown in Figure 1g. This multiscale structure has the potential to capture and scatter sound waves of diverse frequencies, thereby enhancing the absorption effect of wide‐frequency sound waves. Furthermore, the freeze‐drying process facilitates the direct sublimation of frozen water in melamine/MWCNTs solution foam, efficiently preventing the formation of a dense MWCNTs film between the skeleton during conventional drying (Figure S4, Supporting Information). Therefore, this method ensures that the composite foam maintains high porosity and pore connectivity, essential for effective acoustic absorption. In addition, water serves as the solvent in the fabrication process of our composite foam, ensuring safety and environmental friendliness.

**Figure 2:**
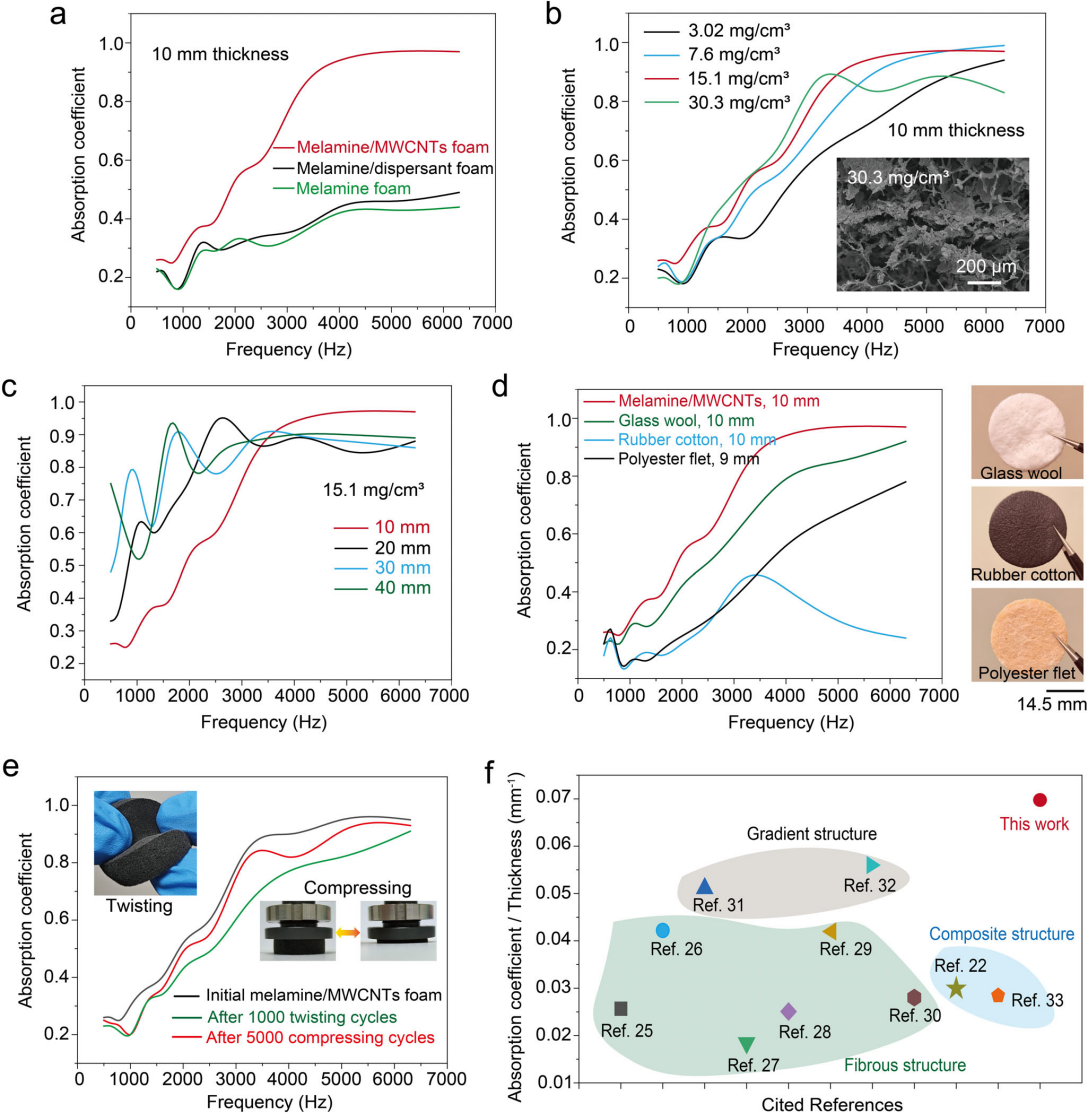
Acoustic absorption performances of the melamine/MWCNTs foam. a) Comparison of the acoustic absorption coefficient for the melamine foam before and after loading MWCNTs, the thickness of all specimens is ≈10 mm. b) Acoustic absorption coefficients of the melamine foams with various MWCNTs content, all maintained ≈10 mm thickness. c) Acoustic absorption coefficient of the melamine foams with various thicknesses, all specimens maintain a constant MWCNT content of 15.1 mg cm^−3^. d) Comparison of acoustic absorption coefficients between the composite foam and commercial acoustic absorbers. e) Stability assessment of our absorber subjected to 5000 compressing/release and 1000 twisting cycles, exhibiting high stability over cycles. f) Comparison of the absorption coefficient‐to‐thickness ratio between melamine/MWCNTs foam and other reported porous absorption materials.

The melamine/MWCNTs foam, characterized by high porosity, through pores, and multiscale pores, presents superior acoustic absorption performances. As illustrated in **Figure** **2**a, its absorption coefficient demonstrates a significant improvement compared to the initial melamine foam at a broad frequency range of 500–6300 Hz. This wide absorption spectrum encompasses common noise frequencies, extending from residential and vocal to traffic and industrial noises. Especially in the high‐frequency band of 4000–6300 Hz, this composite foam presents an absorption coefficient of 95% at ≈10 mm thickness. Although the average absorption coefficient (defined as the average value of the selected range) under 500–1000 Hz is only 26.8%, it represents a 37.2% increase compared with that of initial melamine foam. In addition, in the medium‐ and high‐frequency range (1300–6300 Hz), the average absorption coefficient of the composite foam reaches ≈70%, marking a 196.5% increase relative to the initial melamine foam. It is worth noting that the dispersant in the MWCNTs solution also plays a positive role in enhancing the foam's acoustic absorption coefficient. However, its effect is slight, as indicated by the green line in Figure 2a, which suggests that the primary driver of this improvement is the MWCNTs. The content of loaded MWCNTs is a critical factor influencing the acoustic absorption capacity of the composite foam. As demonstrated in Figure 2b, a low MWCNT content, such as 3.02 mg cm^−3^, results in a reduced average absorption coefficient to 44.9% due to the formation of insufficient multiscale pores within the foam. Conversely, excessive MWCNTs also have an adverse effect on the material's acoustic absorption capacity. For example, when the concentration of MWCNTs is 30.3 mg cm^−3^, the sound absorption performance decreases by 6.7% across the entire tested frequencies compared to the sample with 15.1 mg cm^−^^3^ of MWCNTs. This reason is that the excessive MWCNTs would obstruct the open pores (see the inset of Figure 2b), leading to increased sound wave reflection rather than absorption. In addition, the absorption capability for low‐frequency sound waves can be further improved by increasing the specimen's thickness. As demonstrated in Figure 2c, when the composite foam's thickness increases to 20 mm, the average absorption coefficient at 500–1000 Hz reaches 44.3%, representing a 65.3% increase compared with that of the 10 mm‐thick specimen. It should be noted that, based on our experimental results, increasing the thickness of the composite foams to 30 mm can further enhance the absorption coefficient for low‐frequency sound (500–1000 Hz) to 64%. However, when the thickness of composite foam reaches 40 mm, it does not further improve the absorption coefficient and even results in a slight reduction at high frequencies. The possible reason is that excessive thickness leads to an uneven distribution of pores in the composite foam, affecting the scattering and absorption of sound waves.

Furthermore, we assessed the commercial potential of this composite foam by comparing it with several commercial acoustic absorbers such as glass wool, rubber cotton, and fibrous polyester felt. The test results demonstrated that our melamine/MWCNTs absorber exhibits the highest acoustic absorption coefficient at comparable thicknesses (Figure 2d), suggesting it meets the acoustic absorption requirements for commercial products. Additionally, the adhesion properties of the dispersant and the high mechanical robustness of the commercial melamine foam both ensured stable acoustic absorption performances of the melamine/MWCNTs foam under complex deformations and environments. As illustrated in Figure S5 (Supporting Information), although the gradual mass reduction of the composite absorber attributed to MWCNT detachment as the number of mechanical cycles increased, the overall mass loss remained minimal. The mass reductions were only 3.6% and 7.2% after 5000 compression cycles and 1000 twist cycles, respectively, confirming the high mechanical stability of the composite material. The absorption coefficient of the composite foam showed only minor changes after 1000 twisting cycles and 5000 compressive tests (Figure 2e). Furthermore, the durability of the melamine/MWCNTs foam was assessed through ultrasonic cleaning, with the results illustrated in Figure S6 (Supporting Information). While ultrasonic cleaning is more aggressive to cause MWCNTs detachment compared to compression or twisting tests, the mass loss primarily occurs during the early stages and then tends to stabilize. Correspondingly, the average sound absorption coefficient also exhibits a similar trend, with a significant reduction in the rate of decline after 120 s, suggesting the formation of a stable composite structure. Moreover, the melamine/MWCNTs foam exhibits superior acoustic absorption efficiency compared to reported porous absorbers of the same thickness, including conventional porous materials,^[^ ^25^, ^26^, ^27^, ^28^, ^29^, ^30^ ^]^ gradient porous materials,^[^ ^31^, ^32^ ^]^ and composite aerogels,^[^ ^22^, ^33^ ^]^ as shown in Figure 2f. These results strongly prove that reconstructing pores in a common porous foam by loading MWCNTs is an economic and practical strategy to improve its acoustic absorption efficiency while maintaining initial geometric shape and thickness.

**Figure 3:**
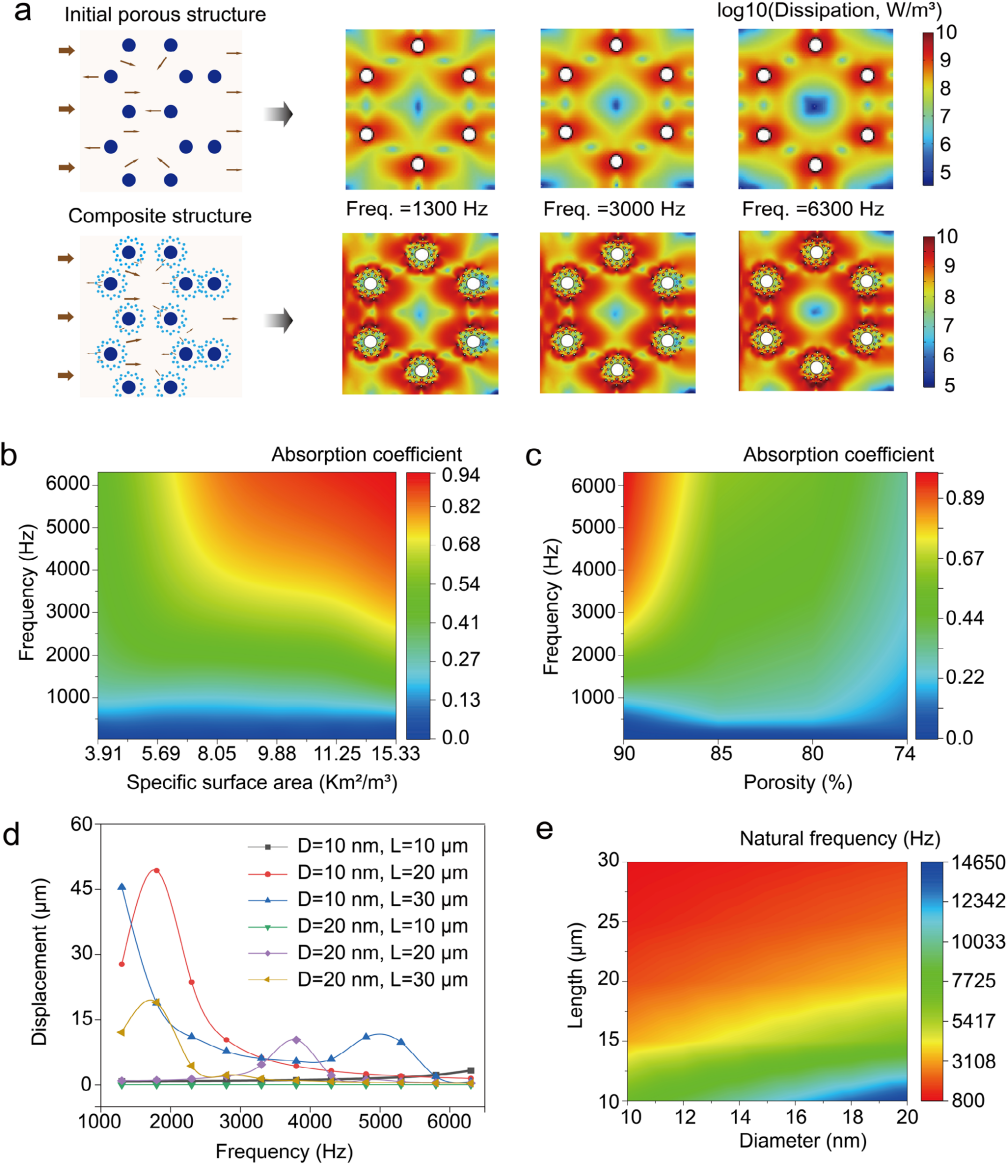
Mechanism of acoustic absorption of the melamine/MWCNTs foam. a) Schematic illustration of sound wave propagation in the pores of a simple and a composite structure, along with their heat maps for thermal dissipation from COMSOL modeled. b) Influence of the specific surface area on the acoustic absorption performance of the porous materials. c) Absorption coefficient of the composite materials with different porosity. d) The vibrational displacement of pristine MWCNTs with various aspect ratios under a frequency range of 1300–6300 Hz. e) Influence of the aspect ratio on the natural frequency of the MWCNTs.

The above results clearly demonstrate that MWCNTs significantly enhance the acoustic absorption of our composite foam. Therefore, it is crucial to investigate and understand the enhancement mechanism to better design high‐performance absorbers. The MWCNTs are linear materials characterized by a nanoscale diameter and a high length‐to‐diameter ratio. When they infiltrate the pores of porous materials, MWCNTs aggregate to form complex micro‐ to nanoscale pore structures and additional boundaries (see the SEM image in Figure 1g), significantly increasing the material's spatial curvature and specific surface area. As a result, the propagation path of sound waves in composite porous materials with a multiscale porous structure becomes more tortuous, leading to increased multiple reflection and airflow friction, which results in greater dissipation of sound energy. **Figure** **3**a presents the heat map of viscous flow dissipation for initial porous structure and composite porous structure at frequencies of 1300, 3000, and 6300 Hz. It is evident from the simulated results that the highest energy dissipation predominantly occurs in the regions surrounding the solid skeletons. Compared to the initial porous foam, the composite foam exhibits a more extensive region of high dissipation energy (indicated by dark brown) across all simulated frequencies. It means that the surface area per unit volume of a porous solid substance is a crucial factor in reducing the sound wave energy. To evidence this point, we investigated the propagation process of sound waves through pore structures with identical porosity (95%) but different specific surface areas. Noted that the unit of specific surface area is set as Km^2^/m^3^ in the context of 2D models. The simulation results clearly indicate a consistent increase in the absorption coefficient as the specific surface area of the porous structure expands under a certain range (Figure 3b), which agrees well with the experimental results. However, the porosity gradually decreases as the composite structure is further filled with microstructures. Lower porosity hinders sound waves penetration into the porous material, reducing their effective conversion into other forms of energy and then lowering the sound absorption coefficient, as illustrated in Figure 3c. Certainly, the absorption coefficient is also influenced by the pore's size in addition to the specific surface area and porosity. An extremely small pore size, such as one less than the mean free path of air (≈65 nm), hinders the efficient transfer of acoustic energy from the air, resulting in inefficient acoustic absorption. Figure S7 (Supporting Information) supports this point, demonstrating that under the same porosity, nanoscale pores lead to a decreased absorption coefficient due to the strong acoustic wave reflection effect by the dense skeletons. Fortunately, our composite structure not only possesses abundant large and interconnected pores from initial melamine foam, but also features a diverse pore size distribution ranging from µm to nm due to MWCNTs accumulation. These large and open pores provide ample space for sound wave penetration, while the smaller pores significantly enhance the specific surface area, improving energy dissipation and acoustic absorption capability. Therefore, it can be inferred that constructing a composite porous structure within common foam is a feasible strategy for enhancing acoustic dissipation, which is consistent with our experimental results.

In addition, ultrafine nanotubes intrinsically possess low flexural stiffness, resulting in bending instability and consequently exhibiting unique vibrational characteristics. In principle, the strong resonant effect of nanotubes can efficiently dissipate sound vibrations and retard their propagation, which is advantageous for improving acoustic absorption performance. As a result, we investigated the resonance behavior associated with the aspect ratio of MWCNTs by FEA (Figure S8, Supporting Information). To ensure consistency with the size parameters of the MWCNTs used in this study, the lengths of simulated nanofibers are set from 10 to 30 µm, and the diameters vary from 10 to 20 nm. We observe that the reduced bending rigidity of slender nanofibers promotes the conversion of external stimuli into resonant deformation (Figure 3d), which is beneficial for energy dissipation. The resonance response of thick and short nanotubes, such as (e.g., *D *= 20 nm; *L *= 10 µm) is insufficient, resulting in reduced vibrational amplitude. Furthermore, the numerical results further demonstrate that the resonant frequency decreases monotonically with an increase in the length‐to‐diameter ratio (Figure 3e). It is worth noting that the simulated fibers exhibit a broad resonant frequency range from 800 to 14650 Hz, effectively covering the relevant frequencies of our test. This observation highlights the capability of ultrafine MWCNTs to resonate across a wide frequency spectrum for sound absorption. Certainly, the resonant behavior of MWCNTs may be inhibited by fiber stacking in practical structures. However, the resonance behavior remains possible, particularly when a significant number of individual fibers protrude outward at the boundary of the MWCNTs stack, as illustrated in Figure S9 (Supporting Information).

Based on the simulation results, the sound absorption mechanism of the MWCNTs‐based porous material can be summarized as follows: First, the interconnected large pores and high porosity inherited from the initial foam allow sound waves to penetrate the material more effectively, reducing surface reflection (particularly for high‐frequency sound waves), and increasing the interaction between sound waves and the material. Second, the loaded MWCNTs create a multiscale pore structure with a large specific surface area, which significantly increases the propagation path of sound waves and enhances their contact frequency within the material. This structural configuration facilitates multiple reflections of sound waves and enhances airflow friction, improving the material's sound absorption across a broader frequency range. Finally, the strong resonance effect of MWCNTs effectively dissipates sound vibrations and reduces their propagation, further enhancing the sound absorption performance of our composite material. Overall, the sound absorption performance of the melamine/MWCNTs foam is determined by its interconnected and multiscale pores, high porosity, and specific surface area, in conjunction with the resonant behavior of MWCNTs.

**Figure 4:**
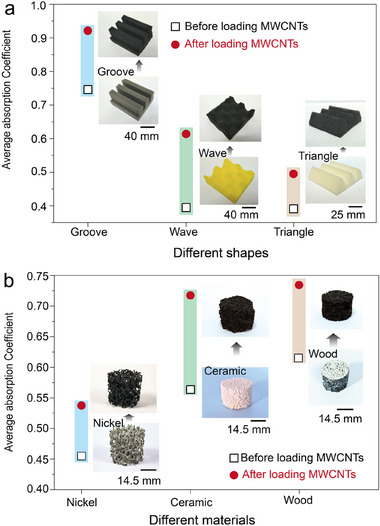
Preparation of various acoustic absorbers by loading MWCNTs in common porous materials. Comparison of the acoustic absorption coefficient and corresponding digital images of porous materials with a) different shapes and b) different raw materials before and after loading MWCNTs.

Furthermore, liquid has a strong capability to uniformly coat curved surfaces owing to its low surface tension. Consequently, reconstructing pores by loading MWCNTs aqueous solution followed by freeze‐drying is a general method suitable for various porous structures, demonstrating robust structural adaptability. For instance, various common acoustic absorption foams with different structures, such as grooves, triangles, and waves, can be modified easily using our method, as illustrated in Figure 4a. The exceptional structural adaptability of this method comes from the unrestricted mobility of MWCNTs solution within pores, even in structures with curved surfaces or 90° corners. In addition, the acoustic absorption coefficient for all structure types showed significant improvement across the entire tested frequency (1300–6300 Hz) after loading MWCNTs. This result strongly supports that reconstructing pores is a practical strategy for addressing the challenges of porous structural configurations in acoustic absorption. Furthermore, this method is applicable in other material systems beyond polymers. Herein, we selected three typical porous materials including ceramic, nickel, and wood, to evaluate the activating effect of loading MWCNTs on their acoustic absorption. Similarly, all tested specimens exhibited an increased absorption coefficient over a broad frequency range. Even for the activated ceramic, the average absorption coefficient could be enhanced to 71.7%, surpassing that of many specially designed absorbers, as illustrated in **Figure** **4**b. The original sound absorption coefficient curves, along with the information on the thickness and MWCNTs content of these specimens, are comprehensively presented in Figure S10 (Supporting Information). These results demonstrate that our method is a general strategy that can be readily applied to other material systems characterized by high porosity and low cost. It is worth pointing out that the method of pore reconstruction by incorporating MWCNTs exhibits certain limitations in the pore size of initial porous materials. To illustrate this, a supporting experiment is conducted using porous membranes with different pore sizes (0.45, 1.2, and 5 µm), as shown in Figure S11 (Supporting Information). The results show that the sound absorption coefficient of all tested specimens increased after incorporating MWCNTs. However, the increments in these porous materials are 26.6%, 26.5%, and 7.7%, respectively, which are significantly lower than those observed in melamine/MWCNTs foam. SEM images reveal that the majority of MWCNTs assemble on the surface of the porous membrane, particularly on smaller pores (such as 0.45 and 1.2 µm), hindering the effective infiltration of MWCNTs solution. Consequently, these materials with smaller pore sizes cannot achieve an efficient pore reconstruction using MWCNTs, unlike commercial melamine foam. Actually, a gradient porous structure is easily formed when MWCNTs are filtered on the membrane surface. Although the porous membrane with 5 µm pore size exhibits higher permeability for the MWCNTs solution, allowing more MWCNTs to penetrate into the membrane, a significant number of MWCNTs still adhere to the membrane surface. This leads to the replacement of initial microscale pores on the membrane surface with smaller pores formed by the accumulation of MWCNTs. These structures cause significant reflection of sound waves at the membrane surface, hindering sound wave penetration into the membrane's interior and reducing sound energy dissipation.

**Figure 5:**
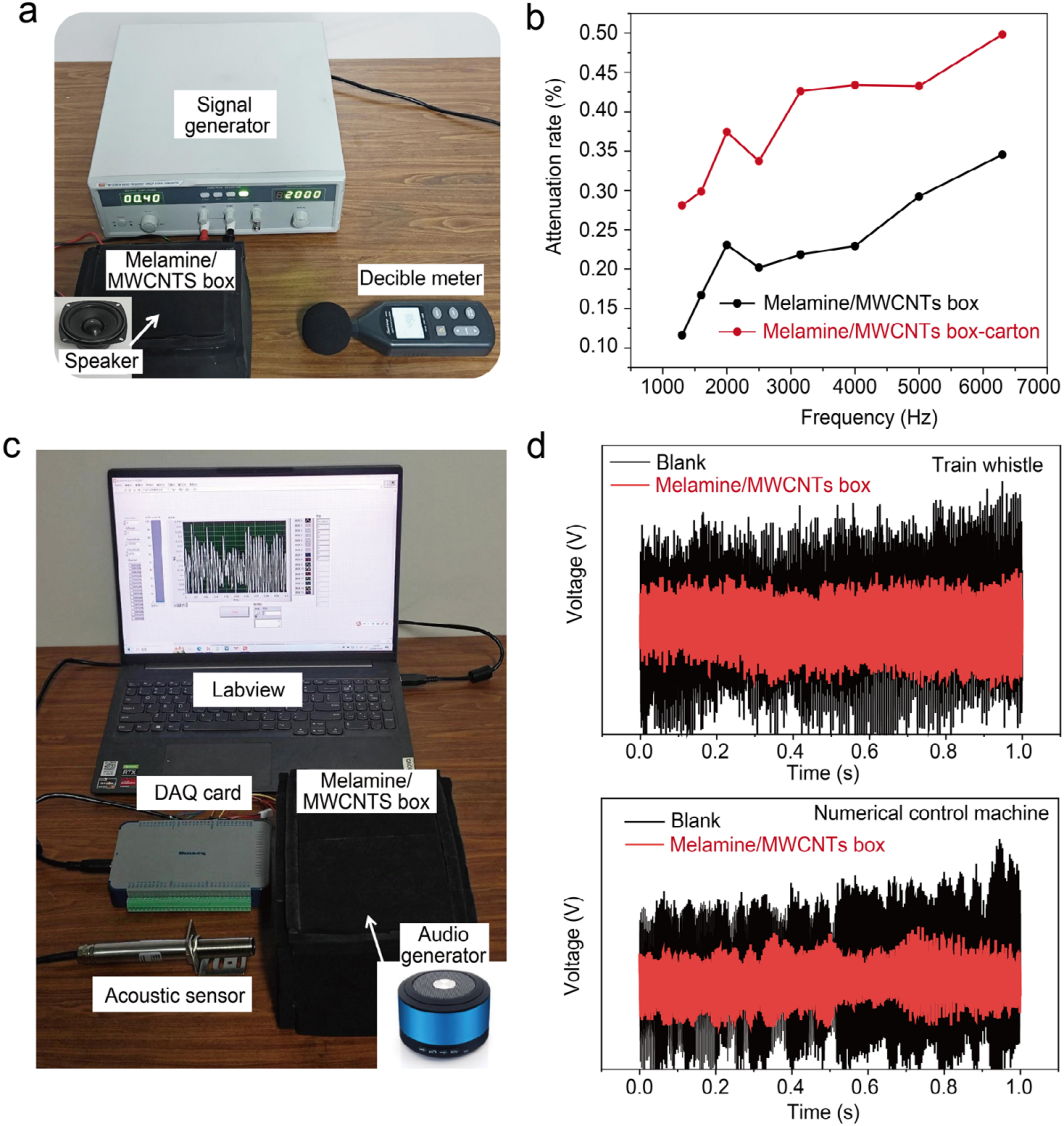
Practical applications of the melamine/MWCNTs composite absorber. a) Illustration of the prototype test setup for composite absorber in practical applications, including signal generator, melamine/MWCNTs boxes, speaker, and decibel meter. b) The attenuation rate for the original sound of melamine/MWCNTs boxes and melamine/MWCNTs boxes‐carton with different frequency sound sources. c) The device of practical acoustic application with complex sound, including an audio generator, melamine/MWCNTs boxes, acoustic sensor, and DAQ card. d) The signal intensity with or without melamine/MWCNTs boxes with audio of “Numerical control machine” and “Train whistle” as sound sources.

The application potential of the composite foam‐based absorber is evaluated through a prototype test. We fabricated an acoustic box using the melamine/MWCNTs foam and placed a PZT buzzer inside the box. The buzzer can generate single‐frequency sounds by converting the alternating current frequency into sound signals. The attenuated sound from the buzzer inside the acoustic box was detected using a decibel meter, with the experimental setup schematically illustrated in **Figure** **5**a. The test results demonstrate that the composite foam‐based enclosure effectively attenuates acoustic signals across a wide frequency range from 1300 to 6300 Hz. The attenuation rate, defined as the ratio of the sound intensity with and without the acoustic box, averaged 22.5% across the full frequency range and the peak value was 34.6%. In addition, the decibel reduction is directly proportional to the frequency of acoustic waves, as illustrated by the black line in Figure 5b. It is consistent with the previously experimental results from acoustic absorption coefficient tests. Furthermore, the noise reduction capability of the composite foam‐based box can be further improved by placing it within a cardboard enclosure to form an acoustic absorption and insulation assembly. The average attenuation magnitude is improved to 49.8%, more than double that achieved with a single composite foam (see the red line in Figure 5b). Furthermore, to assess the absorption efficiency of complex sound by the composite foam, an experimental setup was established as illustrated in Figure 5c. A Bluetooth speaker served as sound source, while a data acquisition card (DAQ) was employed to collect digital signals across a range of acoustic frequencies. Regarding the audio such as “Train whistle” and “Numerical control machine,” a significant reduction is observed in sound intensity when subjected to the composite foam‐based absorber, as shown in Figure 5d. The exceptional sound absorption performance in simulated environments shows that our proposed absorber has significant potential for applications in industrial production, architectural decoration, and traffic facilities.

## 3 Conclusion

In summary, this study provides a general strategy to enhance the acoustic absorption of common porous materials by loading MWCNTs. This approach facilitates the conversion of single large pores into multiscale composite pores, significantly increasing the specific surface area while preserving high porosity and initial thickness. These features significantly increase the probability of interactions between sound waves and porous structures, enhancing the dissipation of sound energy. Meanwhile, the vibration effect of MWCNTs with a high aspect ratio has the potential to further increase the efficiency of energy absorption. The composite foam not only effectively absorbs medium‐ and high‐frequency sound while maintaining high mechanical stability, but also demonstrates strong adaptability to the morphology and raw material of initial porous substrates. This study provides a simple and feasible strategy for fabricating porous acoustic materials with high absorption coefficients, tailored to meet the specific acoustic control requirements of various applications.

## 4 Experimental Section

### Fabrication of Melamine/MWCNTs Composite Foam

The MWCNTs used in this work were purchased from Xianfeng Nanomaterials Technology Co., Ltd. The aqueous solution of MWCNTs was obtained by dispersing MWNCTs into water with the aid of dispersants and ultrasound. The dispersant (XFZ20) was purchased from Xianfeng Nanomaterials Technology Co., Ltd. To ensure the formation of a homogeneous dispersion solution, the weight ratio of MWCNTs to dispersant was set to 1:1. The mixed solution was subjected to ultrasonic treatment using an ultrasonic disintegrator. The power of the disintegrator was set to 700 W, and an intermittent ultrasonic mode was employed (3 s of ultrasound followed by a 3‐s pause). To prevent overheating and subsequent water evaporation, the solution was cooled after every 30 min of ultrasonic treatment. The total ultrasonic treatment duration was 1 h. The composite acoustic absorbers were fabricated by immersing a commercial melamine foam in the dispersion solution of MWCNTs and then removing the water through freeze drying.

### Characterizations and Measurements

Micro‐morphological images of all specimens were obtained by using a field‐emission scanning electron microscope (Gemini300). The acoustic absorption coefficient measurements of all specimens were evaluated using an impedance standing‐wave tube (SZZB) over a frequency range of 500–6300 Hz. All porous polymer samples were precision‐cut into circular specimens with diameters of 29 or 100 mm using a laser cutting machine. The nickel and ceramic foams were procured online with custom diameters. For triangular and trough‐shaped foams, the 29 mm circular specimens fully encompassed their respective structural units (angle structures), ensuring that the periodic characteristics of these structures were preserved in the tested specimens. For wavy foam, due to the larger size of individual structural units, a 29 mm circular specimen could not simultaneously contain both a complete wave crest and trough. To address this, sampling was conducted at the peak position of the wave, ensuring that each specimen included the complete wave crest and more than half of the trough. Notably, the sampling positions for all three types of foam simultaneously captured the highest and lowest points of the structure, thereby preserving their periodic characteristics and ensuring the representativeness of the sampling and experimental comparability. The mechanical performance and cyclic compression of the foam absorber were tested by using a tensile machine (XLD‐100E) at a loading speed of 5 mm min^−1^ under ambient temperature. The application display of the acoustic absorber was implemented using the acoustic sensor (ZTS‐300BK‐ZS‐V05), oscilloscope (TBS1104), data acquisition card (NI USB‐1252A), and decibel meter (SM450).

### Finite Element Analysis

Finite element modeling and the mechanisms of acoustic absorption in melamine/MWCNTs foam were investigated through FEA using the commercial software COMSOL Multiphysics. In general, three mechanisms consistently contribute to the effective sound absorption in the melamine/MWCNTs foam: 1) thermoviscous dissipation of airflow; 2) specific surface area and porosity; 3) structural vibration of MWCNTs. The investigation of these mechanisms was conducted using the Thermoviscous Acoustic module, the Pressure Acoustic module incorporating the Johnson‐Champoux‐Allard (JCA) model, and the Structural Mechanics module, respectively. Further details on the COMSOL models, loading/boundary conditions, and solution setups are included in Supporting Information.

## Conflict of Interest

The authors declare no conflict of interest.

## Author Contributions

J.X. and J.L. contributed equally to this work. Q.L. conceived the research. Q.L. and J.X. designed the experiments and analyzed the data. J.L. conducted the finite element analysis. J.X., W.L., Y.L., and L.L. did the acoustic absorption test of composite foam. J.X. designed the prototype test of the acoustic absorber. Q.L. wrote the manuscript and M.W., G.Z., S.R., X.N., B.B., and Q.W. provided suggestions and feedback.

## Supporting information



Supporting Information

## Data Availability

The data that support the findings of this study are available from the corresponding author upon reasonable request.
