# Supplementary material for: Enhanced Broadband Acoustic Absorption in Commercial Foam via Multiwall Carbon Nanotube‐Induced Pore Reconstruction
Source: Adv Sci (Weinh). 2025 Mar 24;12(19):2501898. doi: 10.1002/advs.202501898 (PMC12097108; doi:10.1002/advs.202501898)
Supplement: Supplementary file 1 — Supporting Information [file ADVS-12-2501898-s001.docx]

**Supporting Information**

**Enhanced Broadband Acoustic Absorption in Commercial Foam via Multiwall Carbon Nanotube-Induced Pore Reconstruction**

*Jinkui Xiong^#1^, Jinlong Liu^#3^, Wengui Lin^1^, Yifei Li^1^, Longchao Liao^1^, Mingfu Wen^1^, Guisheng Zhong^1^, Xiaodong Niu ^1^, Longshi Rao^1^, Quan Wang^4,7^, Bin Bao^2^, Qingxian Liu^1,5,6^**

^1^Department of Mechanical Engineering, Shantou University, Shantou, Guangdong, 515063, China, e-mail: [qxliu@stu.edu.cn](mailto:qxliu@stu.edu.cn)

^2^School of Mechanical Engineering and Automation, Harbin Institute of Technology, Shenzhen, Guangdong, 518055, China

^3^Department of Mechanics and Aerospace Engineering, Southern University of Science and Technology, Shenzhen, Guangdong, 518055, China

^4^Department of Civil and Environmental Engineering, Shantou University, Shantou, Guangdong, 515063, China

^5^Intelligent Manufacturing Key Laboratory of Ministry of Education, Shantou University, Shantou, Guangdong, 515063, China

^6^Shantou Key Laboratory for Intelligent Equipment and Technology, Shantou University, Shantou, Guangdong, 515063, China

^7^College of Engineering, Eastern Institute of Technology, Ningbo, Zhejiang, 315000, China

^#^ Jinkui Xiong and Jinlong Liu contributed equally to this work.

*To whom correspondence should be addressed. Email: [qxliu@stu.edu.cn](mailto:qxliu@stu.edu.cn)


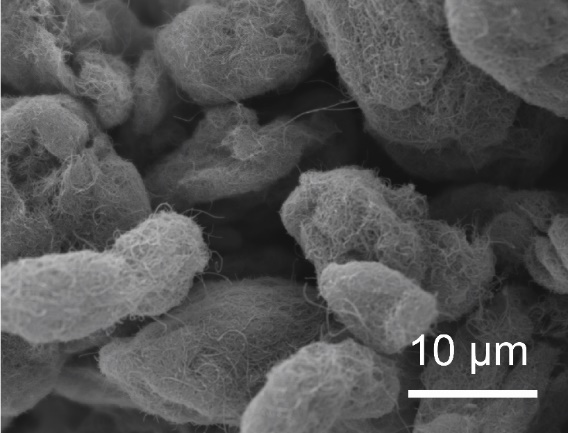


**Figure S1**. SEM image of the agglomerated MWCNTs formed in water without dispersant. Due to strong Van der Waals forces and significant entanglement effects, MWCNTs with high aspect ratio tend to aggregate and form clusters. Such aggregations have potential to block the pores of substrate foam, adversely affecting the acoustic absorption performance of the final composite foam.


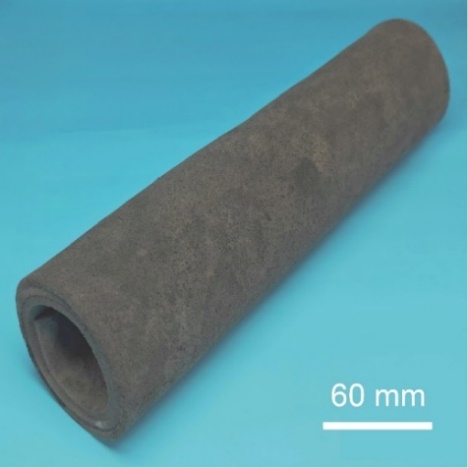


**Figure S2.** A large-scale melamine/MWCNTs foam measuring 10 × 25 cm was fabricated by rolling the sample into a cylindrical shape followed by freeze-drying. It demonstrates significant potential for the method of reconstructing pore structures, thereby facilitating large-scale production.


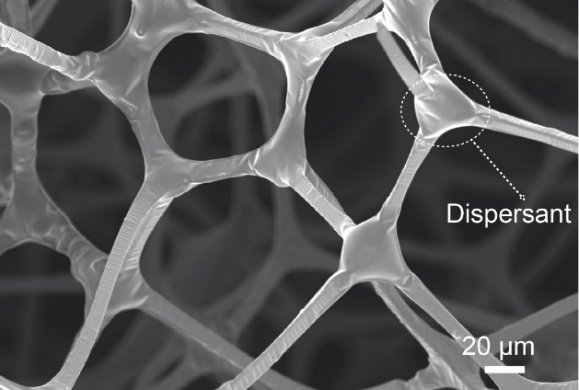


**Figure S3.** SEM image of the melamine/dispersant foam. It clearly shows that the dispersant only coats the surface of the melamine skeletons without filling the pores, thereby largely preserving the original porous structure after drying.


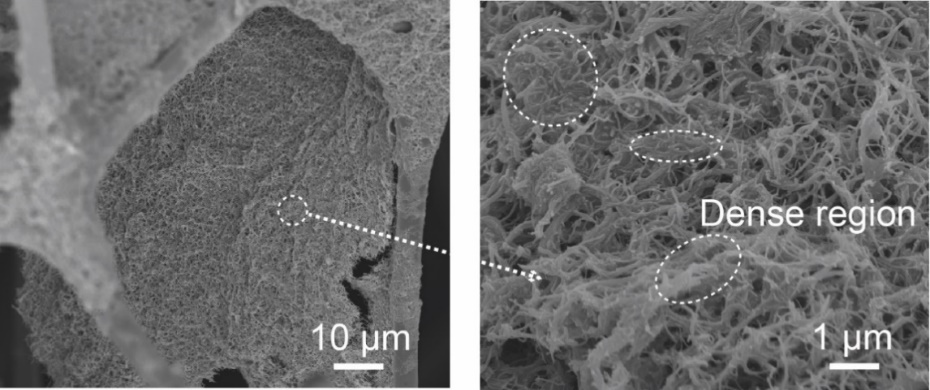


**Figure S4.** SEM image of the melamine/MWCNTs foam fabricated by natural drying. The images reveal that some pores of the initial melamine foam were partially blocked by the MWCNTs film. Meanwhile, many regions of the MWCNTs film exhibit a dense packing, which is likely to restrict the deeply penetration of sound waves.


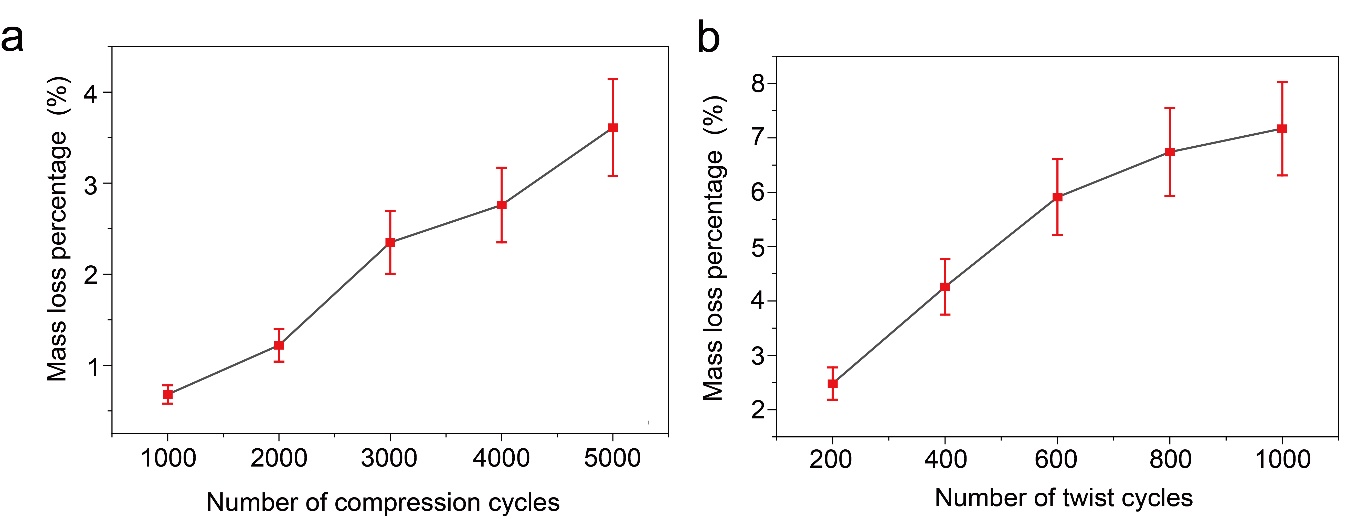


**Figure S5.** Mass change percentage of the melamine/MWCNTs foam after (**a**) compression cycles and (**b**) twist cycles. Although the composite absorber exhibited a gradual mass reduction with increasing cycle numbers, the overall mass loss remained minimal, with only 3.6% and 7.2% reduction observed after 5000 compression cycles and 1000 twist cycles, respectively. These results indicate that the MWCNTs form strong interfacial bonds with the melamine foam matrix, resulting in a highly stable composite sound absorber with remarkable mechanical durability.


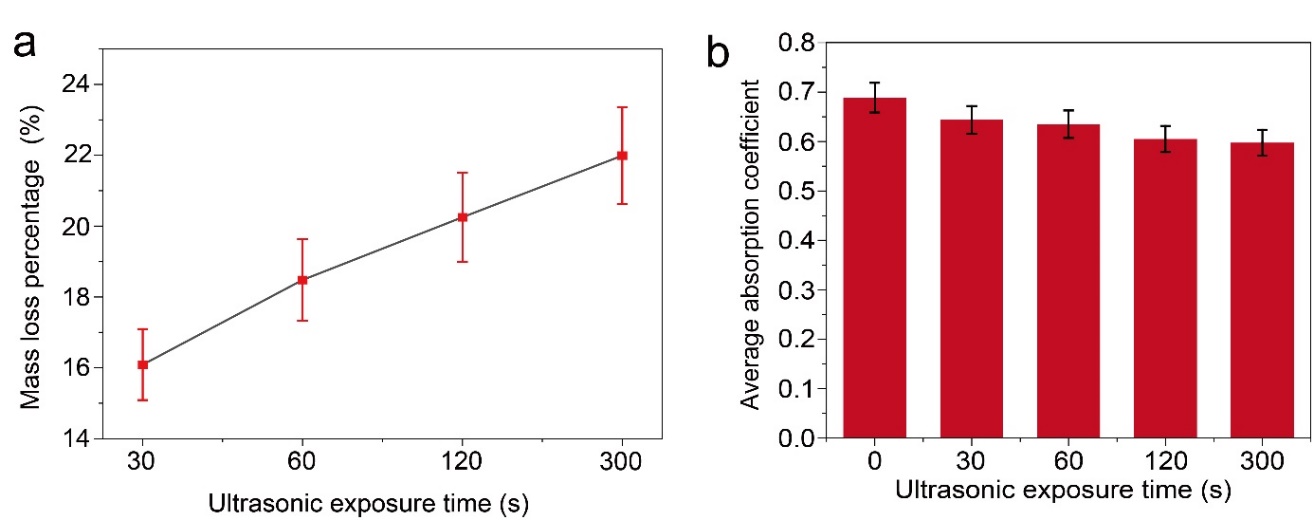


**Figure S6.** Durability test of the melamine/MWCNTs foam by ultrasonic cleaning. (**a**) The mass change ratio of the melamine/MWCNTs foam as a function of ultrasonic exposure time at 24 W power. The results indicate that the mass loss, primarily due to the progressive detachment of MWCNTs, reaches up to 16.1% after 30 seconds of ultrasonic cleaning. This demonstrates that ultrasonic cleaning is more destructive compared to compression or twisting tests. Notably, most of the mass loss occurs during the initial stages of ultrasonic cleaning and then tends to plateau. (**b**) The average sound absorption coefficient of the melamine/MWCNTs after ultrasonic cleaning. It also exhibits a similar gradual decrease, aligning with the shedding of MWCNTs. However, it stabilizes after 120 seconds, suggesting the formation of a stable composite structure.


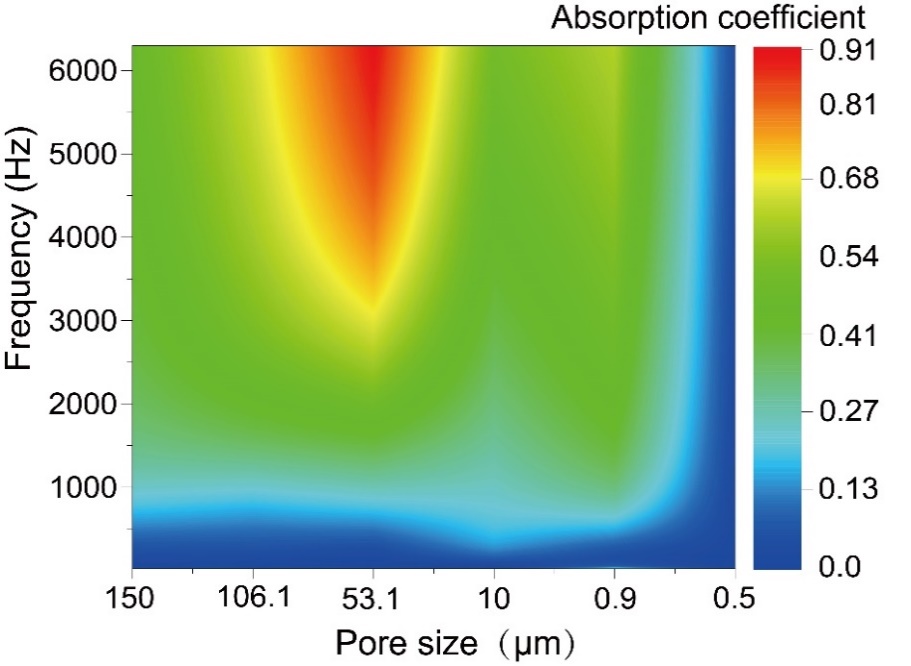


**Figure S7.** Influence of pore size on the acoustic absorption performance. Oversized pores reduce the acoustic absorption coefficient because sound waves pass through without being absorbed. Nanoscale pores also exhibit an adverse impact as they significant prevent sound waves from penetrating deeply enough into the material and are immediately reflected back into the surrounding air.


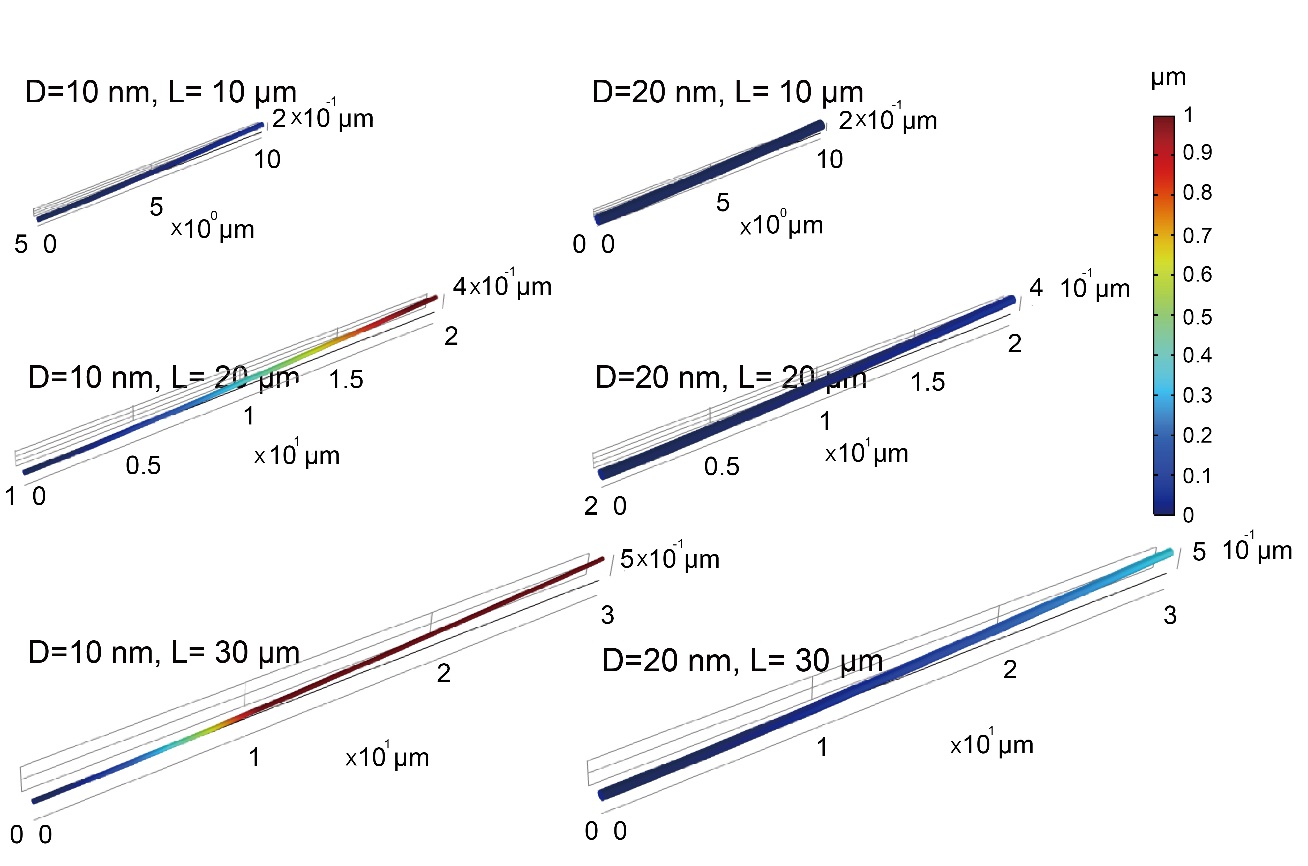


**Figure S8.** The simulation of vibrational displacement of the MWCNTs with various aspect ratios over a wide frequency range of 800 to 6000 Hz. Results indicate that MWCNTs with a smaller diameter and a longer length contribute to achieving deformation under sound field conditions, thereby enhancing energy dissipation of sound waves.


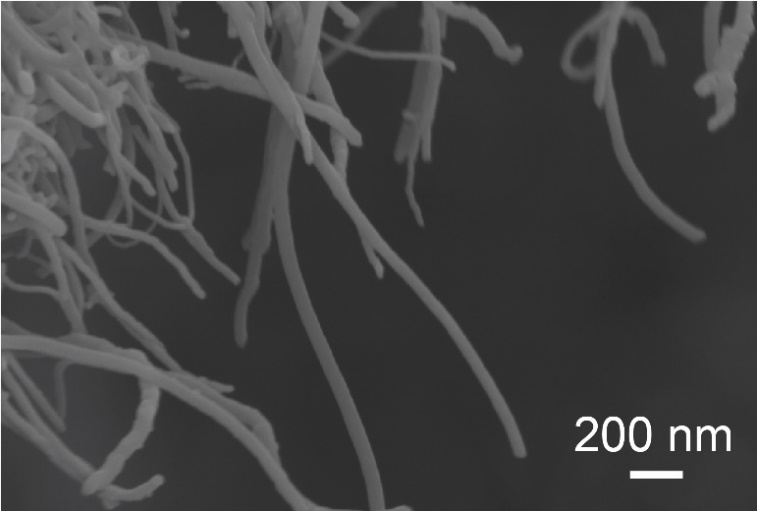


**Figure S9.** SEM image of single MWCNTs situated at the periphery of the MWCNTs layer. These MWCNTs have capable to vibrate in response to sound waves, converting sound energy into mechanical energy and enabling effective absorption of sound waves.


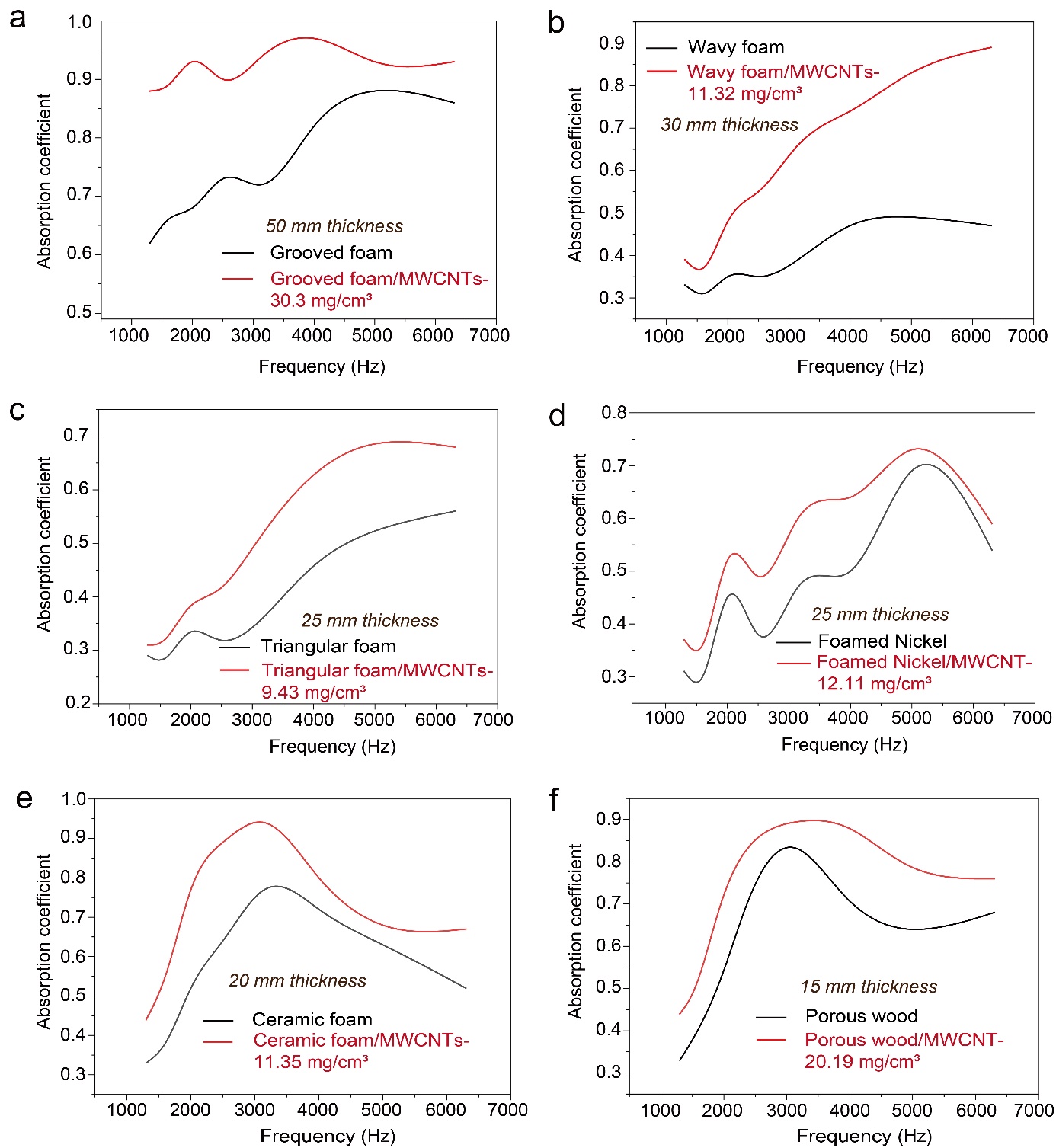


**Figure S10.** The original sound absorption coefficient curves for specimens with distinct configurations: (**a**) groove, (**b**) wave, and (**c**) triangle in shape, and (**d**) nickel, (**e**) ceramic, and (**f**) wood in raw materials. It should be noted that the thickness of the specimens and the content of MWCNTs vary across each sample type, with detailed information provided in the corresponding images.


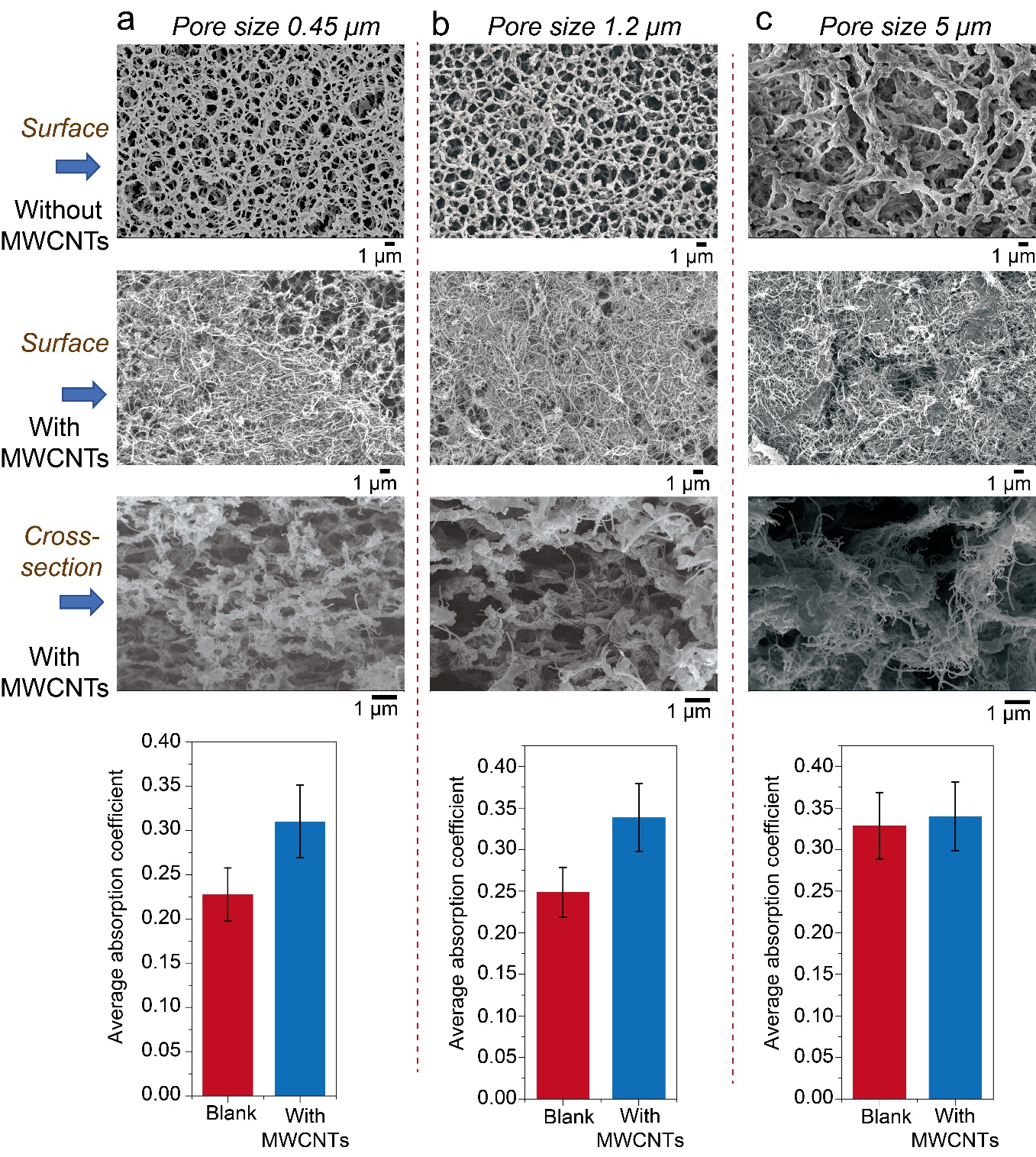


**Figure S11.** The effect of the pore size in initial material for the pore reconstruction and sound absorption performance. (**a-c**) the microstructures and sound absorption coefficients of porous materials with commercially marked pore sizes of (**a**) 0.45 μm, (**b**) 1.2 μm, and (**c**) 5 μm before and after loading MWCNTs. It should be noted that the actual observed pore size in these materials may differ from their commercially marked dimensions. Nevertheless, the experimental results clearly indicate that smaller pore sizes impede MWCNT permeation into the porous materials, leading to significant surface accumulation and consequently affecting pore reconstruction.

**Numerical Simulations:**

1. Thermoviscous dissipation of airflow:

To achieve a trade-off between computational accuracy and efficiency, a cut plane of the porous foam along the direction of sound wave propagation was utilized for modeling. To enhance the modeling efficiency at the microscale, only a unit cell and the critical components that influence the underlying physics were retained. Therefore, the solid skeletons and MWCNTs were modeled as circles. Arranged in a regular pattern, both the initial and composite porous structures were represented using circles with diameters varying from 10 nm to 30 μm. The pore structures, including porosity, pore size, and specific surface area, were controlled by adjusting the diameter of the solid skeletons and their spacing. In contrast to the solid components, referring to the solid skeletons and MWCNTs, modeling was done with just the air phase.

The Thermoviscous Acoustic module was adopted, utilizing the boundary layer theory for modeling acoustic viscous and thermal losses. Plane-wave radiation was then applied to both the upstream and downstream boundaries of the computational domain. The no-slip condition, where the airflow speed is set to zero, was applied to the boundaries of the solid skeletons and MWCNTs. The periodic boundary condition (PBC) was applied to the rest boundaries of the computational domain. Besides, the initial pressure, velocity, and temperature variation were all set as zero. To derive the view of heat maps, the thermos-viscous dissipation was demonstrated using the built-in variable of “ta.diss_tot”.

1. Specific surface area and porosity:

The numerical investigation of specific surface area and porosity effects on the performance of the melamine/MWCNTs foam should be carried out in the following two steps:

1. Derive the macroscopic acoustic parameters: The finite element modeling utilized to construct porous structures with different specific surface areas and porosities was analogous to that of the initial porous structure. In this case, the pore structures, including porosity, pore size, and specific surface area, were controlled by adjusting the diameter of the solid skeletons and their spacing.

The Johnson-Champoux-Allard (JCA) model in the pressure acoustic module was adopted to investigate the macroscopic acoustic response of the melamine/MWCNTs foam. The JCA model was recognized as one of the most effective semi-phenomenological models, where the dynamic density and dynamic bulk modulus were defined using five parameters: the porosity $\phi$, flow resistance $\sigma$, the high-frequency tortuosity $\alpha_{\infty}$, viscous characteristic length $\Lambda$, and thermal characteristic length $\Lambda^{'}$. The porosity φ and thermal characteristic length $\Lambda^{'}$ can be calculated directly by definition. The rest parameters can be obtained by solving two steady-state boundary value problems posed on a fluid domain:

1. $\sigma$ was derived by solving a Stokes flow problem driven by a unit pressure gradient. The pressure inlet and outlet were applied to the upstream and downstream boundaries of the computational domain, respectively. The no-slip condition was applied to the boundaries of the solid skeletons. The symmetric boundary condition was applied to the rest boundaries of the computational domain.
2. $\alpha_{\infty}$ and $\Lambda$ were derived by solving a potential flow problem. The velocity inlet and outlet were applied to the upstream and downstream boundaries of the computational domain, respectively. The no-penetration condition was applied to the boundaries of the solid skeletons. The symmetric boundary condition was applied to the rest boundaries of the computational domain.

2. Derive the acoustic absorption coefficient: To achieve better computational efficiency, a two-dimensional rectangular computational domain was adopted. The whole domain was divided into two parts: air and porous foam subdomains. For the air subdomain, plane wave radiation was applied to the upstream boundary. The sound hard boundary was applied to the two lateral boundaries adjacent to the upstream boundary. For the porous foam subdomain, the macroscopic acoustic parameters derived in the previous step were required as input for the JCA model. Subsequently, the acoustic absorption coefficients associated with various frequencies were derived.

**Calculation formula of the five macroscopic acoustic parameters:** including porosity ($\phi$), flow resistivity ($\sigma$), tortuosity ($\alpha_{\infty}$), viscous characteristic length ($\Lambda$) and thermal characteristic length ($\Lambda^{'}$).

$\phi=\frac{V_{air}}{V_{total}}$ (1)

$\sigma=\frac{1}{d}\cdot\frac{\Delta p}{u}$ (2)

$\alpha_{\infty}=\frac{L}{d}=\frac{\left\langle u^{2} \right\rangle}{\left\langle u \right\rangle^{2}}$ (3)

$\Lambda=2\frac{\int_{\Omega_{air}} u^{2}dV}{\int_{\partial\Omega_{air}} u^{2}dS}$ (4)

$\Lambda^{'}=2\frac{\int_{\Omega_{air}} dV}{\int_{\partial\Omega_{air}} dS}$ (5)

$\left\langle* \right\rangle=\frac{1}{\Omega_{air}}\int_{\Omega_{air}} \left( * \right)dV$ (6)

In formula (1), $V_{air}$and $V_{total}$represent the volume of air and the total volume of the porous material, respectively. In formula (2), $d$ represents the thickness, $\Delta p$ is the pressure difference between the two sides of the porous material, and $u$ represents the fluid velocity perpendicular to the surface of the porous material; In formula (3), $L$ and $d$ represent the actual propagation path length and material thickness of the sound wave in the porous material, respectively, and $u$ represents the propagation speed of the sound wave. In formula (4) and (5), $\Omega_{air}$represents the volume domain of the hole, $\partial\Omega_{air}$represents the surface area of the hole; In formula (6), $\left\langle* \right\rangle$ is the mean value of the variable * is calculated in the air domain of the porous material.

**Table S1.** Five parameters corresponding to models with different specific surface areas.

| Specific surface area（${m^{2}}/{m^{3}}$） | Porosity | Flow resistivity（$Pa*s/m^{2}$） | Tortuosity | Viscous characteristic length (μm) | Thermal characteristic length (μm) |
| --- | --- | --- | --- | --- | --- |
| 3905.9 | 0.95 | 8710 | 1.016 | 262.96 | 512.43 |
| 5692 | 0.95 | 17480 | 1.016 | 178.81 | 351.37 |
| 8053 | 0.95 | 35140 | 1.016 | 126.68 | 248.34 |
| 9877.4 | 0.95 | 52910 | 1.016 | 103.51 | 202.48 |
| 11245 | 0.95 | 70000 | 1.016 | 91 | 177.85 |
| 15332 | 0.95 | 124000 | 1.016 | 66.85 | 130.44 |

**Table S2.** Five macroscopic parameters corresponding to models with different porosity.

| Porosity | Flow resistivity（$Pa*s/m^{2}$） | Tortuosity | Viscous characteristic length (μm) | Thermal characteristic length (μm) |
| --- | --- | --- | --- | --- |
| 0.9 | 110130 | 1.0342 | 27.6 | 59.2 |
| 0.85 | 1020900 | 1.0477 | 16.11 | 33.372 |
| 0.8 | 1310400 | 1.0684 | 10.08 | 21.17 |
| 0.74 | 4376600 | 1.1075 | 5.704 | 12.92 |

1. Structural vibration of MWCNTs:

To explore the aspect ratio effect on resonance vibration, MWCNTs were modeled as cantilever beams with various geometric parameters (D = 10~20 nm, L = 10~30 μm) in the Structural Mechanics module. The natural frequencies of the MWCNTs with varying aspect ratios were determined through eigenfrequency analysis. Subsequently, the vibration responses of the MWCNTs within the investigated frequency range (from 1300 to 6300 Hz) were analyzed through the forced vibration analysis. As with the mechanical tests, the input material properties include the Young’s modulus of 0.3 GPa, density of 1101 kg/m^3^, and Poisson’s ratio of 0.44.
